# Quantitative TEM imaging of the magnetostructural and phase transitions in FeRh thin film systems

**DOI:** 10.1038/s41598-017-18194-0

**Published:** 2017-12-19

**Authors:** Trevor P. Almeida, Rowan Temple, Jamie Massey, Kayla Fallon, Damien McGrouther, Thomas Moore, Christopher H. Marrows, Stephen McVitie

**Affiliations:** 1 SUPA, School of Physics and Astronomy, University of Glasgow, Glasgow, G12 8QQ UK; 2 School of Physics and Astronomy, University of Leeds, Leeds, LS2 9JT UK

## Abstract

Equi-atomic FeRh is a very interesting material as it undergoes a magnetostructural transition from an antiferromagnetic (AF) to a ferromagnetic (FM) phase between 75–105 °C. Its ability to present phase co-existence separated by domain walls (DWs) above room temperature provides immense potential for exploitation of their DW motion in spintronic devices. To be able to effectively control the DWs associated with AF/FM coexistence in FeRh thin films we must fully understand the magnetostructural transition and thermomagnetic behaviour of DWs at a localised scale. Here we present a transmission electron microscopy investigation of the transition in planar FeRh thin-film samples by combining differential phase contrast (DPC) magnetic imaging with *in situ* heating. We perform quantitative measurements from individual DWs as a function of temperature, showing that FeRh on NiAl exhibits thermomagnetic behaviour consistent with the transition from AF to FM. DPC imaging of an FeRh sample with HF-etched substrate reveals a state of AF/FM co-existence and shows the transition from AF to FM regions proceeds via nucleation of small vortex structures, which then grow by combining with newly nucleated vortex states into larger complex magnetic domains, until it is in a fully-FM state.

## Introduction

Equiatomic iron-rhodium (Fe_48_Rh_52_ to Fe_56_Rh_44_) has attracted considerable attention due to its magnetostructural transition from its antiferromagnetic (AF) to ferromagnetic (FM) phase^1^. This ordered *α*’ alloy adopts a CsCl structure and undergoes a first-order phase transition from its room-temperature AF state to FM between ~75 to 105 °C, and can hence present phase AF/FM co-existence and hysteresis^2^. The co-existing phases are separated by a phase-boundary domain wall (DW) and effective control over the creation and motion of these phase-boundary DWs are considered desirable for potential application in a new generation of novel nanomagnetic or spintronic devices^3^. Previous studies have shown that the DWs can be created and driven in FeRh films by combining heating with differential gradients of chemical doping^4,5^. However, our knowledge of the dynamic behaviour of DWs in FeRh is often limited to bulk magnetic measurements or low magnification imaging (resolution in the order of 10 s of nm), *i.e*. magnetic force microscopy^6,7^, x-ray magnetic circular dichroism (XMCD)^7^. For example, XMCD photoelectron emission microscopy (PEEM) has been used to observe the phase coexistence in FeRh thin films and to show the first order transition from the nucleation of domains regime to be distinct from the domain growth regime^4,8^. The magnetostructural transition has also been followed *in situ* through scanning electron microscopy with polarisation analysis (SEMPA) and suggested that the interfacial ferromagnetism coexisting with the AF phase inside the film is an intrinsic property of the FeRh (001) surface^9^. Nevertheless, these techniques are often limited to a spatial resolution of ~20–30 nm in typical cases^10,11^ and ~5 nm for SEMPA in specialised SEM instruments^12^, as well as being restricted in penetration depth to a few nm^10,13^. These experimental limitations prevent imaging of the localised dynamic evolution of the aformentioned nucleation and growth stages of the magnetostructural transition, as well as precise quantitative analysis from individual domains. Hence, in order to fully understand the magnetostructural transition and dynamic motion of DWs in FeRh thin films, it is necessary to investigate their associated mechanisms at an even more localised scale through their entire thickness, whilst applying external stimuli, *i.e. in situ* heating.

Significant advances in knowledge are potentially enabled using the transmission electron microscopy (TEM) techniques of Fresnel imaging^14,15^, off-axis electron holography^16–19^ and differential phase contrast imaging (DPC)^20–22^, which allow for the visualising of magnetic induction down to ~1 nm spatial resolution. Electron holography has been combined with *in situ* cooling and heating to investigate the magnetisation of a cross-sectional FeRh thin film, revealing an inhomogeneous spatial distribution of the transition temperature along the growth direction, as well as a regular spacing of the nucleated FM domains^23^. However, preparation of cross-sectional TEM lamellae is inherently destructive and the thermomagnetic behaviour of the thinned FeRh samples is not representative of their original two-dimensional (2D) magnetic state, with shape anistropy becoming a more dominant factor. Indeed, high quality 2D planar view magnetic thin films have been produced for magnetic imaging within the TEM^24^. Further, the development of TEM holders which include micro-electro-mechanical systems (MEMS) has led to the ability to apply a range of external stimuli to samples *in situ* within the TEM, *i.e*. temperature, electrical bias, etc., whilst maintaining stability and minimising drift^25^. Only until very recently, limited work had been reported on the preparation of planar magnetic films on MEMS chips for *in situ* TEM studies^26^. Accordingly, combining high-resolution (HR) magnetic imaging in the TEM with newly developed *in situ* methods can provide fundamental insight into the intricate details of the magnetostructural transition in planar-view FeRh thin films.

The scanning TEM (STEM) technique of DPC imaging permits down to ~1 nm spatial resolution imaging of magnetic induction within nanostructured thin films as a function of applied electric and magnetic fields, as well as temperature^27^. This is the only technique that is presently able to provide both HR images of DW motion in thin magnetic films *in situ* within the TEM and quantitative measurements of spatial induction variation from the DWs, directly. In this study, we perform several STEM techniques to examine the localised chemical, structural and magnetic properties of FeRh films grown epitaxillay grown on MgO substrates or NiAl buffer layers. Cross-sectional and planar FeRh samples have been prepared from bulk substrates by focused ion beam (FIB) methods or HF-etching of the substrates. Comparison is made between the integrity of the planar FeRh TEM samples prepared via different methods and the ability to perform quantitative measurements of magnetic induction from DWs using DPC imaging, as a function of temperature. Here we show the evolution of magnetic domains and the subsequent AF/FM phase co-existence in a FeRh film during *in situ* heating, and mechanisms of the AF to FM phase transition are proposed.

## Results

A series of ordered *α*’-FeRh alloy thin films were grown epitaxially on a clean (001) MgO substrate or (001) NiAl buffer layer on GaAs substrate by conventional DC magnetron sputter co-deposition, as described previously^28^. Cross-sectional and planar FeRh TEM samples were prepared from their bulk substrates and transferred onto *in situ* heating (DENSsolution Wildfire^TM^) electronic (e−) chips by Ga^+^ FIB methods^26^. An additional planar FeRh TEM sample was prepared through a process of HF-etching of AlAs, GaAs and NiAl buffer layers, as well as GaAs substrate^29^, and subsequently transferred onto Cu TEM grids for the purpose of *in situ* heating using a Gatan heating TEM holder. The variation in FeRh samples is aimed at exploring the most suitable FeRh/substrate combination for TEM examination of the magnetostructural transition. The reasoning for the selection of samples is: MgO is a commonly-used substrate for FeRh growth; NiAl can act as a conductive pathway with a long-term view for potential device construction; and the HF-etched sample provides a large planar FeRh thin film without the presence of a substrate. For simplicity, the samples are labelled as follows:

- Sample 1: FeRh thin film on MgO substrate (cross-sectional and planar TEM samples);
- Sample 2: FeRh thin film on NiAl buffer layer/GaAs substrate (cross-sectional and planar TEM samples);
- Sample 3: HF-etched FeRh thin film (planar TEM sample).

**Figure 1:**
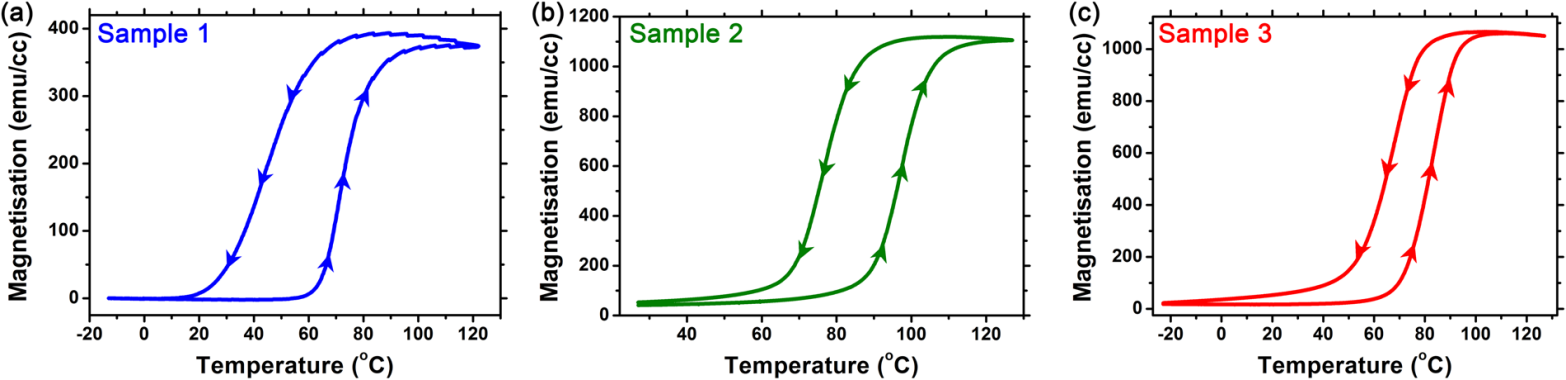
(**a**–**c**) Magnetisation (*M*) vs. temperature (*T*) plots for the FeRh thin films in samples 1–3, respectively.

To provide an overview of the magnetostructural transition in the FeRh thin films, Fig. 1 presents SQUID Vibrating Sample Magnetometer measurements of samples 1–3 as a function of temperature. Sample 1 exhibits a sharp increase in magnetisation from the AF phase at ~60 °C to the FM phase ~370 emu/cc at ~100 °C, with a noticeable asymmetrical hysteresis curve, where the reduction of magnetisation occurs at ~65 °C on cooling. The onset of the transition in Sample 2 occurs at a slightly higher temperature of ~75 °C and stabilises ~1120 emu/cc at ~115 °C, whilst exhibiting a more symmetrical hysteresis curve, with reduction in magnetisation initiating at ~90 °C on cooling. Sample 3 exhibits an increase in magnetisation at ~60 °C and stabilises ~1060 emu/cc at ~100 °C, with a ~20 °C difference in the symmetric hysteresis curve on cooling. All three samples present hysteresis, as expected with a first-order transition, but it is noteworthy that the FeRh samples synthesised on NiAl buffer layers (samples 2 & 3) exhibit a small moment in the AF state, as well as a significantly higher magnetisation in the FM state (~1100 emu/cc) compared to the FeRh on MgO (sample 1, ~380 emu/cc). It is also considered that since the FeRh thin films in samples 1 and 2 are clamped to their respective substrates, the change in lattice parameter of the FeRh is more reliant on the thermal expansion of the MgO substrate or NiAl buffer layer. This produces a larger hysteresis compared to the FeRh thin film in sample 3, which is less restricted to provide a thermomagnetic response.

**Figure 2:**
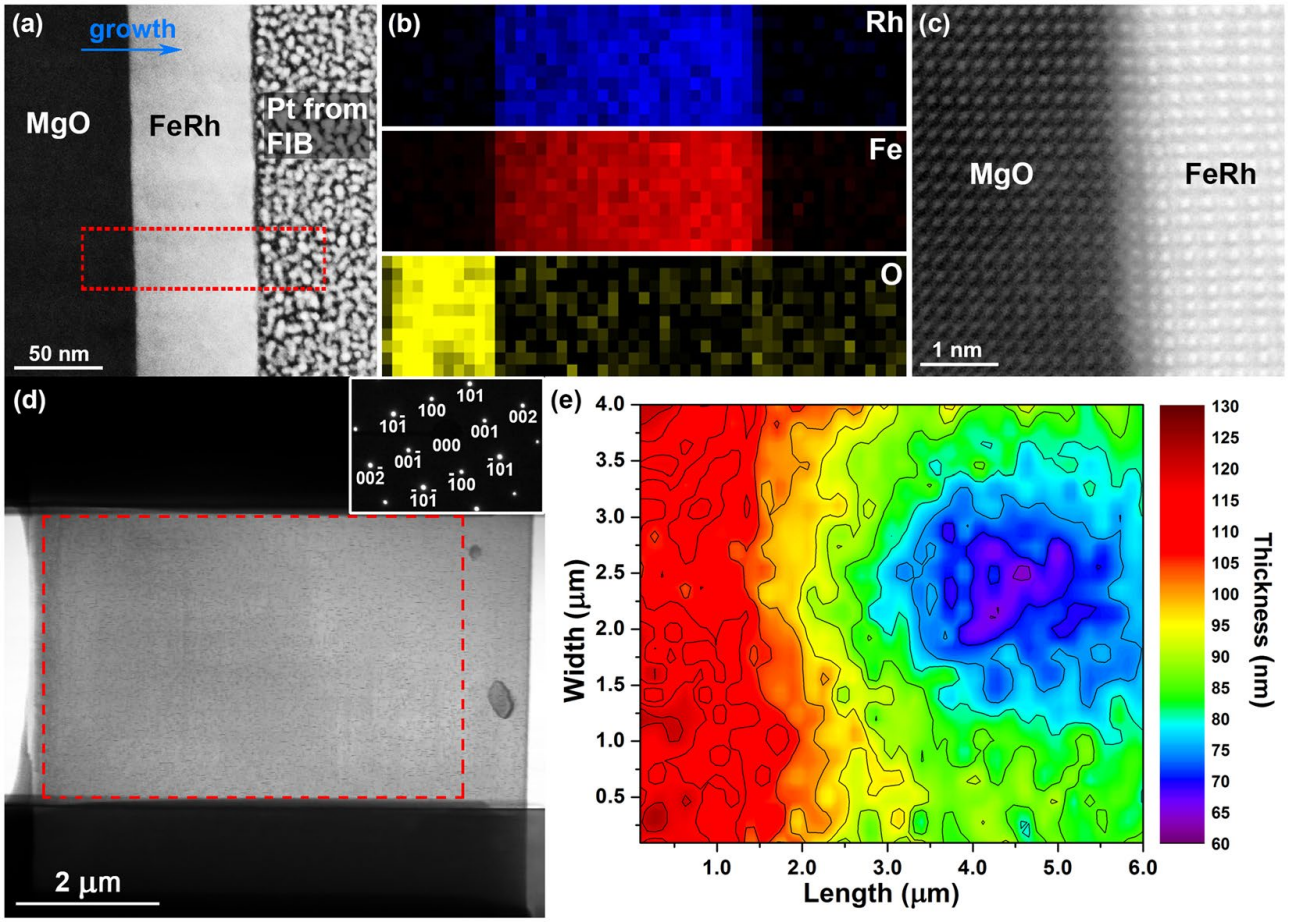
(**a**) DF STEM image of a cross-section of sample 1, showing the FeRh thin film grown on the MgO substrate. (**b**) EELS chemical maps acquired from the box region in (**a**) displaying the elemental distribution of rhodium, iron and oxygen. (**c**) HR STEM image showing the localised structure of the epitaxially grown FeRh and its interface with the MgO substrate. (**d**) BF TEM image of the planar view of sample 1, with the SAED inset. (**e**) Thickness map of sample 1 calculated from the low-loss EEL spectrum acquired from the boxed region in (**d**).

Figure 2 presents a cross-sectional and planar view of the FeRh thin film in sample 1, providing information on its thickness, chemical distribution and interface with the MgO substrate. The dark-field (DF) STEM image of Fig. 2a reveals the FeRh film to be grown with a uniform thickness of ~53 nm, whilst the electron energy-loss spectroscopy (EELS) chemical maps (Fig. 2b) acquired from the boxed region (red) in Fig. 2a display the elemental distribution of rhodium, iron and oxygen. The HR STEM image of Fig. 2c presents the interface between the single crystalline FeRh and MgO substrate, revealing their well-matched orientation and confirming the epitaxial growth of the deposited FeRh. A planar view of sample 1 is shown in the bright-field (BF) TEM image of Fig. 2d, displaying an electron transparent region ~7 µm long and ~4 µm wide. The selected area electron diffraction (SAED) pattern (Fig. 2d, inset) confirms the FeRh film is single crystalline and grown epitaxially on the MgO substrate. Figure 2e presents the thickness map acquired from the boxed region (red) in Fig. 2d, calculated from the low-loss EELS spectrum imaging, where the relative thickness ranges from ~125 nm on the left-hand side to ~65 nm on the centre of the right-hand side.

**Figure 3:**
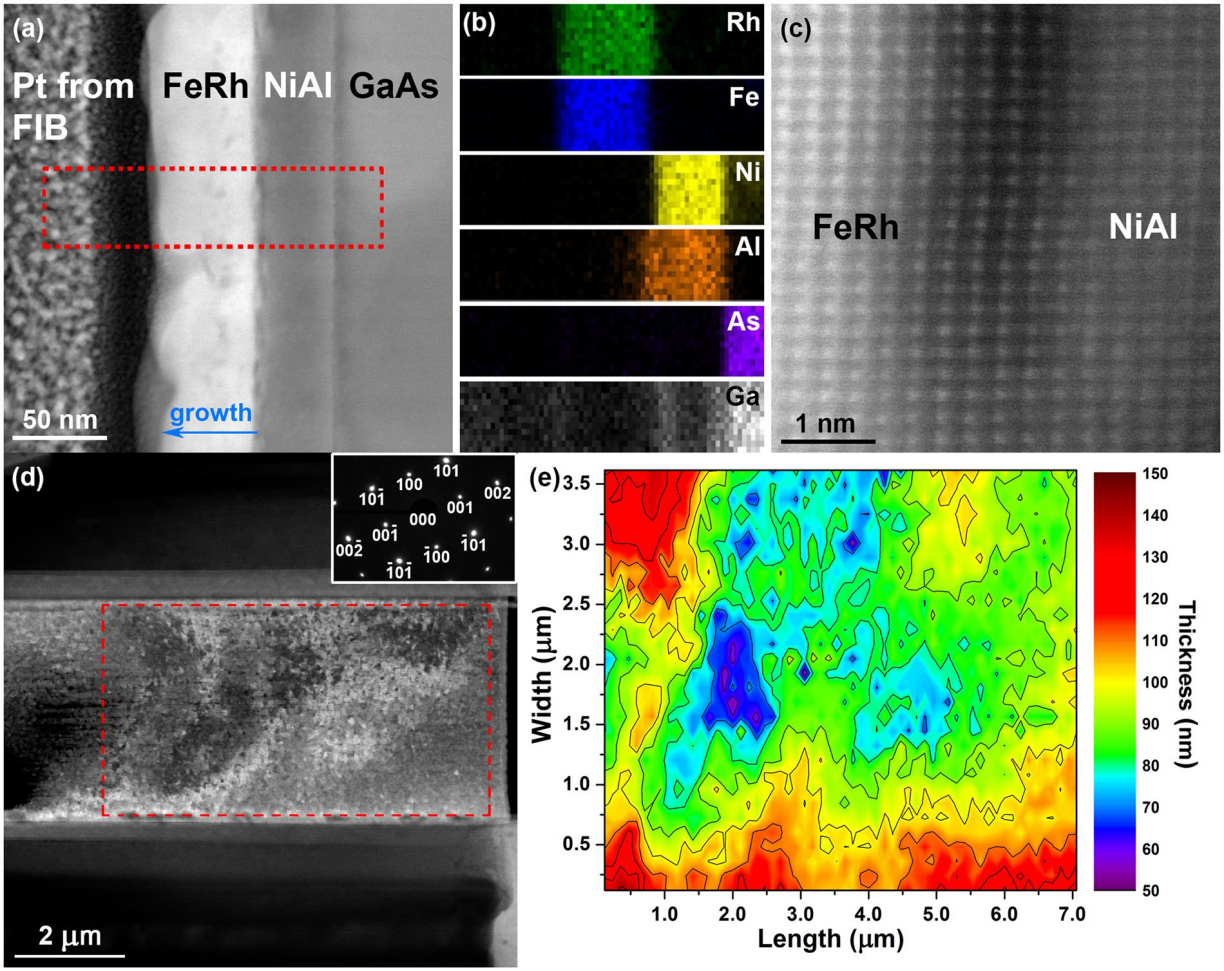
(**a**) DF STEM image of a cross-section of sample 2, showing the FeRh thin film grown on the NiAl buffer and GaAs substrate. (**b**) EDX chemical maps acquired from the box region in (**a**) showing the elemental distribution of rhodium, iron, nickel, aluminium, arsenic and gallium. (**c**) HR STEM image showing the localised structure of the epitaxially grown FeRh and its interface with the NiAl buffer layer. (**d**) DF TEM image of the planar view of sample 2 revealing some mottled contrast, with the SAED inset. (**e**) Thickness map of sample 2 calculated from the low-loss EEL spectrum acquired from the boxed region in (**d**), with thickness variation coinciding with mottled contrast in (**d**).

In a similar fashion to Fig. 2, Fig. 3 presents a cross-sectional and planar view of the FeRh thin film in sample 2. The DF STEM image of Fig. 3a reveals the FeRh film to be grown with a comparatively non-uniform thickness (average of ~50 nm) on the NiAl buffer layer (~40 nm thick) and GaAs substrate. The energy dispersive X-ray (EDX) spectroscopy chemical maps (Fig. 3b) acquired from the boxed region (red) in Fig. 3a display the elemental distribution of rhodium, iron, nickel, aluminium, arsenic and gallium. Figure 3c presents a HR STEM image of the interface between the single crystalline FeRh and NiAl buffer layer, again confirming the epitaxial growth of the deposited FeRh. A planar view of sample 2 is shown in the DF TEM image of Fig. 3d, displaying an electron transparent region ~9.5 µm long and ~4 µm wide that exhibits relatively mottled contrast in comparison to sample 1. Again, the SAED pattern (Fig. 3d, inset) confirms the FeRh film is single crystalline and grown epitaxially on the NiAl substrate. Figure 3e presents the thickness map acquired from the boxed region in Fig. 3d, where the relative thickness ranges from ~135 nm at top left and bottom edges, to ~60 nm on the centre of the left-hand side, and coincides well with the mottled contrast seen in Fig. 3d.

**Figure 4:**
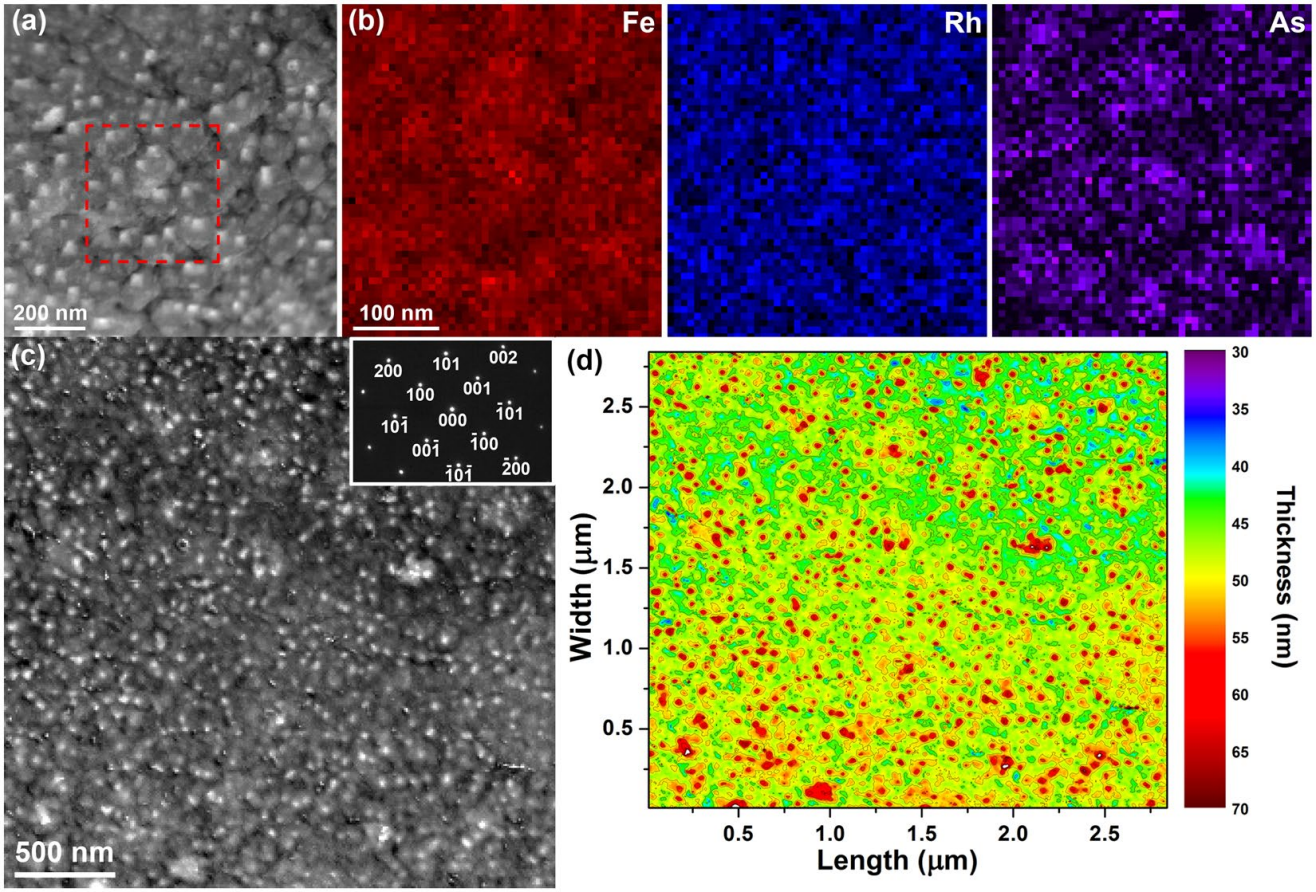
(**a**) DF STEM image of the HF-etched planar FeRh thin film (sample 3). (**b**) EDX chemical maps acquired from the box region in (**a**) showing the distribution of iron, rhodium and arsenic. (**c**) DF STEM image of sample 3 showing small white spots dispersed randomly across the thin film, with the SAED (inset). (**d**) Thickness map of sample 3 calculated from the low-loss EEL spectrum acquired from the entire area of (**c**), with small regions of large thickness coinciding with the spots seen in (**c**).

Figure 4 presents a planar view of the HF-etched FeRh thin film (sample 3), providing details of its surface, morphology, chemistry and relative thickness. The DF STEM image of Fig. 4a reveals the FeRh thin film to exhibit a non-uniform surface and morphology, with variations in contrast attributed to an inconsistent thickness and debris on the surface. The EDX chemical maps (Fig. 4b) acquired from the boxed region (red) in Fig. 4a display the elemental distribution of iron, rhodium and arsenic, revealing a relatively uniform distribution of rhodium and iron, along with concentrated areas of arsenic. The arsenic-rich areas coincide well with the surface debris in Fig. 4a and are considered to be caused by the HF-etching process. Figure 4c presents a DF image of a large square area (~2.8 µm x ~ 2.8 µm) of sample 3 and the SAED pattern (Fig. 4c, inset) confirms the HF-etched FeRh film is single crystalline. Figure 4d presents the thickness map acquired from the entire region of Fig. 4c, where the relative thickness ranges from ~65 nm at the red spots, thought to be arsenic-rich debris dispersed randomly across the thickness of the sample, to a more uniform thickness of ~45–50 nm.

**Figure 5:**
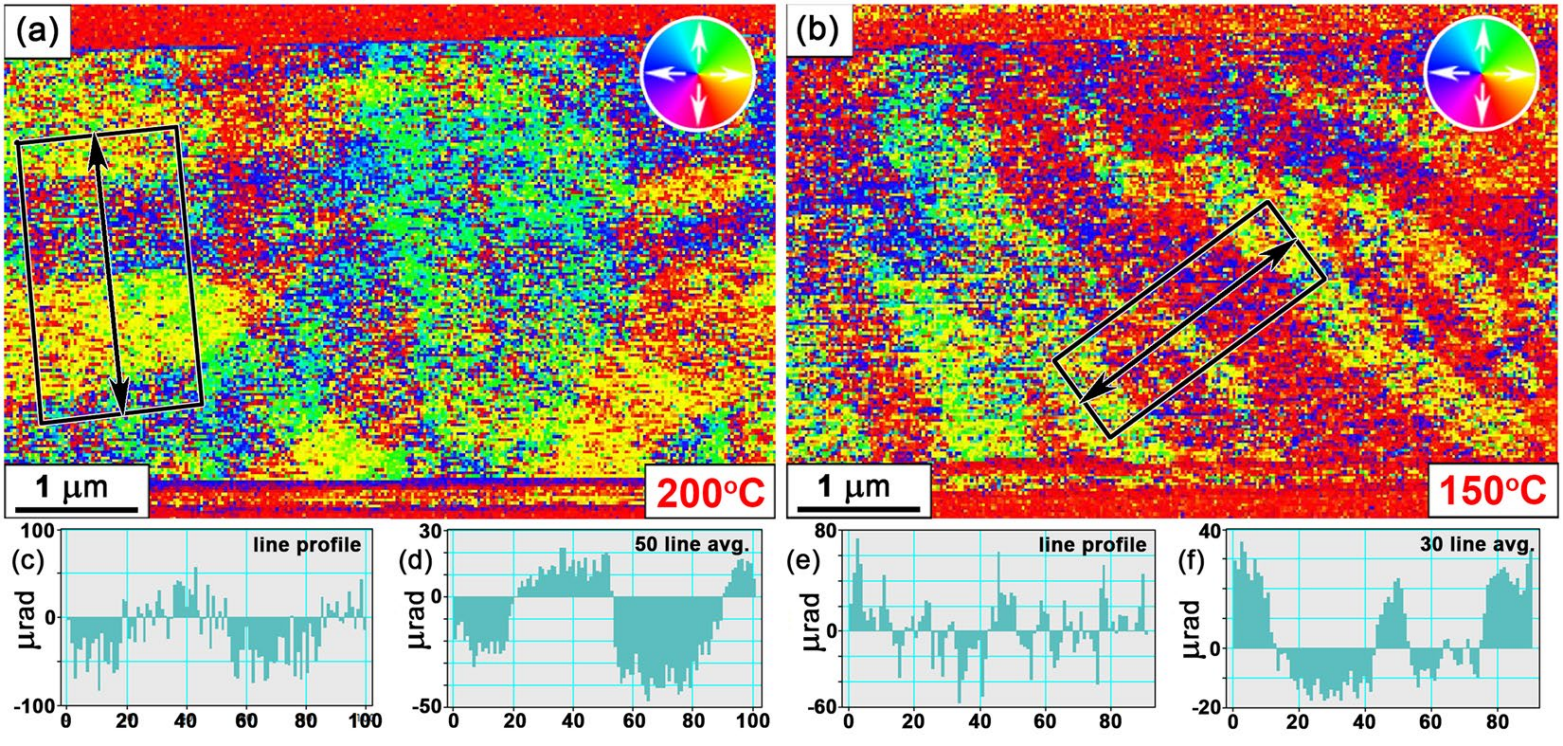
(**a**,**b**) DPC imaging of the planar FeRh thin films when heated *in situ* to (**a**) 200 °C (Sample 1); and (**b**) 150 °C (Sample 2). The direction of magnetisation is depicted in the colour wheels (inset). (**c–f**) Measurements of *β*_*l*_ taken across (**c**,**e**) line and (**d**,**f**) average profiles along the arrows and boxed regions (black) in (**a**) and (**b**).

Figure 5 presents DPC images acquired from planar TEM views of sample 1 and sample 2 during *in situ* heating, providing information of magnetic domain structures of the FeRh thin films. The DPC image of sample 1 heated to 200 °C (Fig. 5a) displays its intricate magnetic domain structure with direction of magnetic induction denoted by the colour wheel (inset). Similarly, the DPC image of Fig. 5b shows the magnetic domain structures of sample 2 heated to 150 °C. In both cases, the magnetic domain structures present are observed to be relatively complicated, with the magnetic contrast considered to include contributions from thickness variations and diffraction contrast, making direct quantitative measurements of magnetic induction challenging. This is demonstrated by the noisy line profiles (Fig. 5c,e) and average profiles (Fig. 5d,f) of the local deflection angle of the electron beam, *β*_*l*_, acquired from the black outlined regions in Fig. 5a,b.

**Figure 6:**
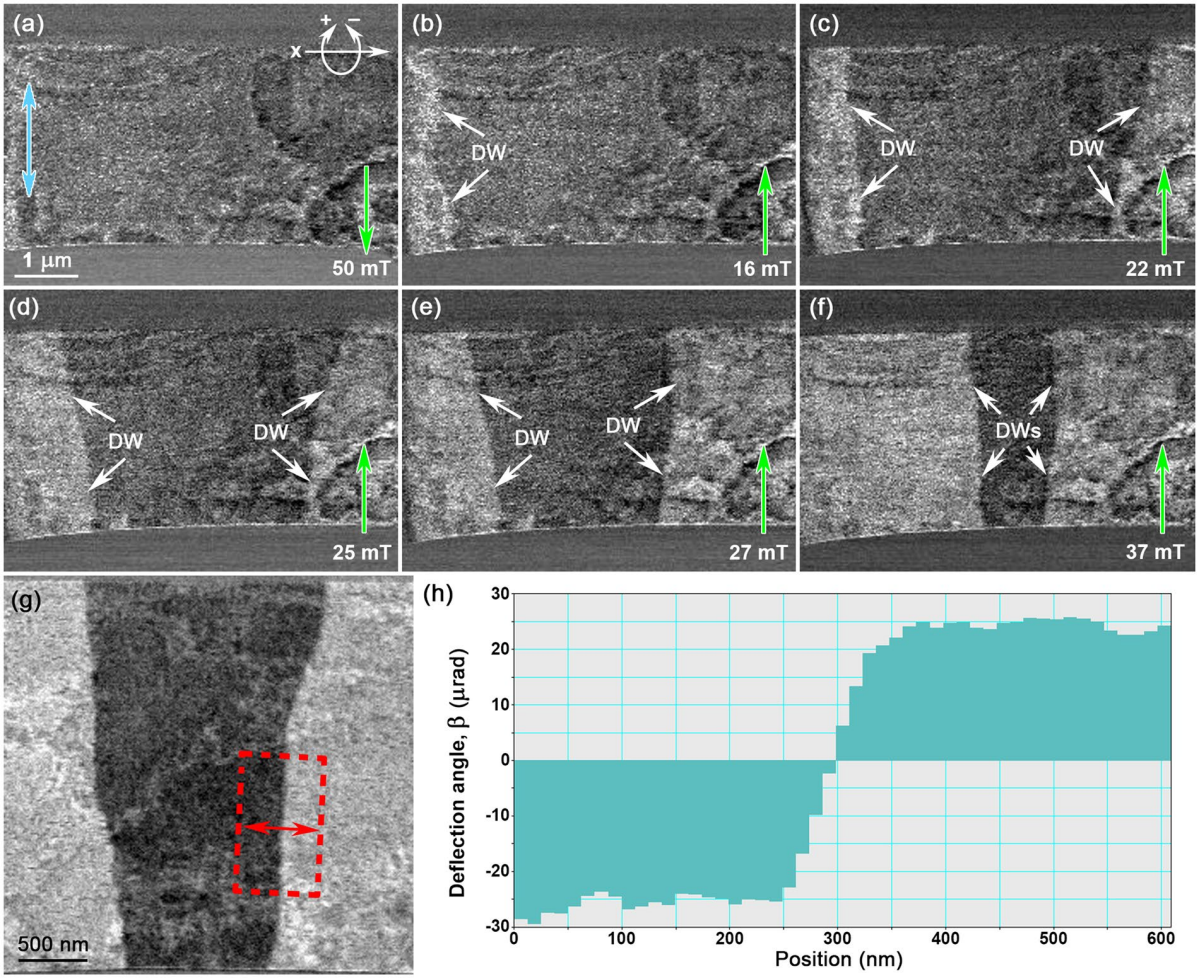
(**a**) DPC image of sample 1 in planar view after *x*-tilting to +23° (shown in top left) and applying an in-plane magnetic field of 50 mT (arrowed, green), and then *x*-tilting to −23°. (**b**–**f**) DPC images of sample 1 in planar view *x*-tilted to −23° and applying in-plane magnetic fields of (**b**) 16 mT; (**c**) 22 mT; (**d**) 25 mT; (**e**) 27 mT; and (**f**) 37 mT (arrowed, green), inducing the movement of the DWs (arrowed, white). (**g**) DPC image of DWs used for measurement of *β*_*l*_ in the form of (**h**) average line profile taken across the boxed region (red) in (**g**). All DPC images are sensitive to the component of magnetic induction indicated by the double-headed blue arrows in (**a**).

In order to aid quantitative measurements, Fig. 6 presents a series of DPC images of sample 1 in planar view at 200 °C and demonstrates the ability to drive domain walls using the magnetic field of the objective lens whilst in the FM phase. For the purpose of accurate measurements, all DPC images of Fig. 6 are sensitive to the component of magnetic induction indicated by the double-headed blue arrow in Fig. 6a. The DPC image of Fig. 6a shows sample 1 after tilting to +23° in the *x*-direction (rotation around the TEM sample rod axis, inset) and applying an in-plane magnetic field of 50mT (labelled by the green arrow) using the objective lens, and then removing the magnetic field and tilting to −23° about the *x*-direction. The tilt angle is changed from +23° to −23° in order to apply an in-plane component magnetic field in the opposite direction (and hence the green arrow faces the opposite direction in Fig. 6b–f). Figure 6b displays nucleation and movement of a DW on the left-hand side (arrowed) under application of an in-plane field of 16 mT at −23° in the *x*-direction. Nucleation of an additional DW can be seen on the right-hand side of sample 1 when the in-plane field is increased to 22mT (Fig. 6c). As the strength of the in-plane field is increased to 25 mT, 27 mT and 37 mT (Fig. 6d–f, respectively), the DWs are driven towards the centre of the planar sample and well-resolved, parallel DWs considered desirable for quantitative analysis are observed (Fig. 6f). The DPC image of Fig. 6g presents parallel DWs with straight regions (red box) that measurements of deflection angle, *β*_*l*_, can be made in the form of average line profiles (Fig. 6h). The deflection angle, *β*_*l*_, across the DW can then be used to calculate the integrated induction, *B*_s_ *t*, in the magnetic domains using the equation:

1 $${B}_{s}t=\frac{{\beta }_{l}h}{e\lambda }$$

where *B*_s_ is the saturation induction, *t* is the thickness of the magnetic thin film (measured from their cross sectional TEM lamellae), *h* is Plancks constant, *e* is the magnitude of electronic charge and *λ* is the electron wavelength. Note that we assume the film has the same structure through its thickness; hence *B*_s_ *t* ∝ *β*_*l*_.

**Figure 7:**
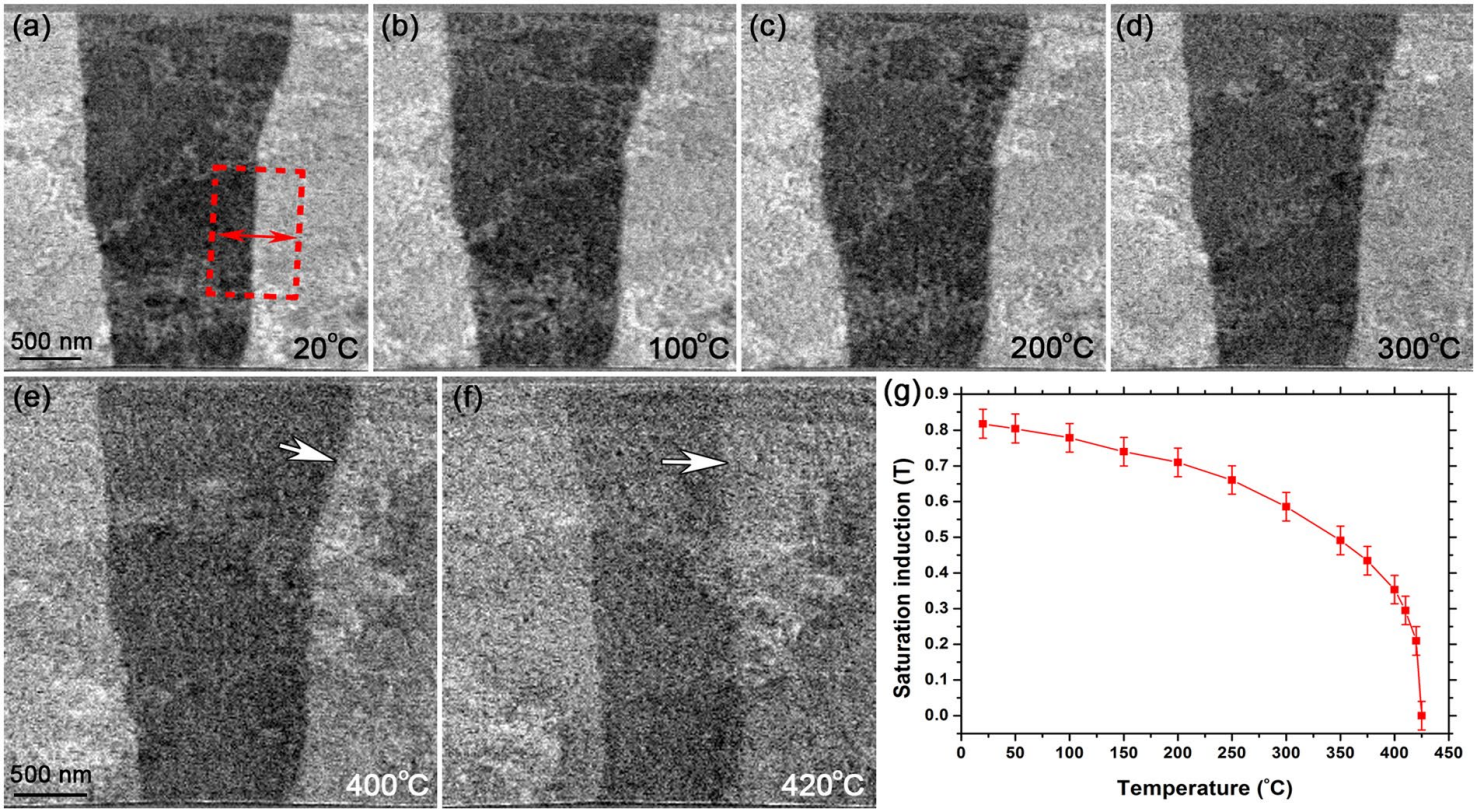
(**a**–**f**) DPC images of magnetic domains/DWs in sample 1 at (**a**) 20 °C; and during *in situ* heating to (**b**) 100 °C; (**c**) 200 °C; (**d**) 300 °C; (**e**) 400 °C; and (**f**) 420 °C. (**g**) Graph of saturation induction as a function of temperature, calculated using direct measurements of *β*_*l*_ from the DW in the boxed region (red) defined in (**a**).

Figure 7 presents the thermomagnetic behaviour of magnetic domains/DWs in sample 1 as a function of temperature. The DPC image of Fig. 7a shows well-resolved DWs at 20 °C and for the purpose of quantitative measurements a 100-line average profile was measured across the right-hand side DW (boxed region, red). Successive measurements were taken during *in situ* heating at 100 °C, 200 °C and 300 °C (Fig. 7b–d). Figure 7e reveals a noticeable decrease in contrast between the magnetic domains at 400 °C, suggesting a loss of spontaneous magnetic induction with temperature. The DPC image of Fig. 7f exhibits a change in position of the right-hand side DW compared to Fig. 7e (arrowed), and indicates a thermally-induced movement of the DW as the FeRh thin film approaches its *T*_*C*_. Figure 7g presents the saturation induction of sample 1 as a function of temperature, calculated using equation (1) and *β*_*l*_ measured from the same DW (boxed region in Fig. 7a) at temperature intervals between 20 °C to 425 °C. The error bars denote a standard deviation of ±5% in the measured *β*_*l*_ values and was calculated directly from the line profiles using Digital Micrograph. At this point, it is worth noting that the thermomagnetic behaviour of sample 1 shown in Fig. 7g is not consistent with its corresponding SQUID measurements from the entire thin film of sample 1 (Fig. 1a). This is considered to be due to the TEM sample preparation and is addressed in more detail in the Discussion.

**Figure 8:**
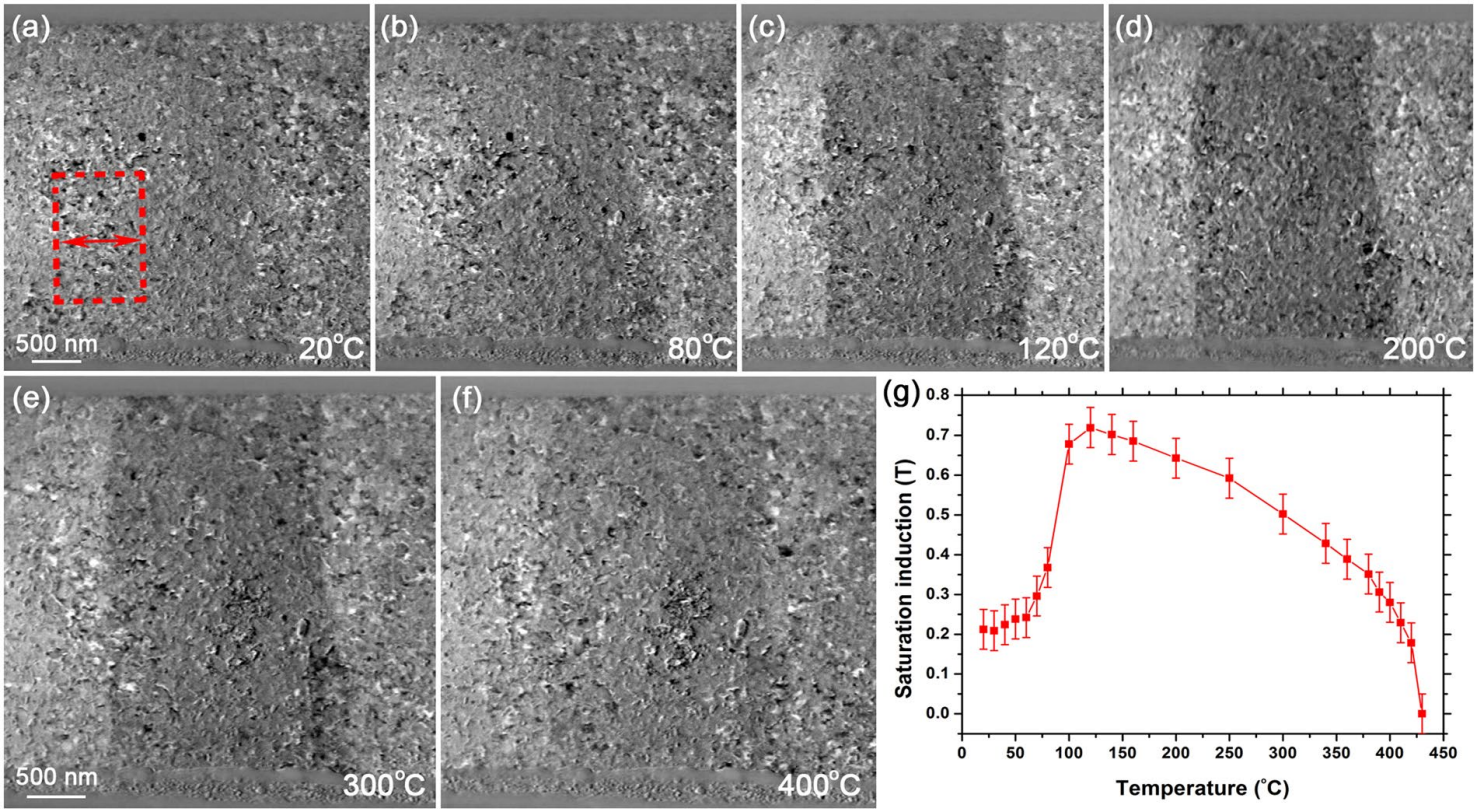
(**a**–**f**) DPC images of magnetic domains/DWs in sample 2 at (**a**) 20 °C; and during *in situ* heating to (**b**) 80 °C; (**c**) 120 °C; (**d**) 200 °C; (**e**) 300 °C; and (**f**) 400 °C. (**g**) Graph of saturation induction as a function of temperature, calculated using direct measurements of *β*_*l*_ from the DW in the boxed region (red) defined in (**a**).

Similarly to Fig. 7, Fig. 8 presents the thermomagnetic behaviour of the magnetic domains/DWs in a plan view of sample 2 as a function of temperature. Figure 8a displays a DPC image acquired at 20 °C, which in the bulk sample would be in the AF phase (Fig. 1b), and the DWs are observed to be markedly noisy compared to sample 1 (Fig. 7a). Nevertheless, a 100-line average profile was also measured across the right-hand side DW (boxed region, red). The DPC image of Fig. 8b exhibits a slight increase in contrast during *in situ* heating to 80 °C, which then becomes more pronounced in Fig. 8c–e during heating to 120 °C, 200 °C and 300 °C, respectively. Figure 8f reveals a subsequent decrease in contrast at 400 °C, again suggesting a loss of spontaneous magnetic induction as the FeRh thin film approaches its *T*_*C*_. Using the same methodology as Fig. 7g, Fig. 8g presents the saturation induction of sample 2 as a function of temperature, using direct measurements from the DPC imaging at temperature intervals between 20 °C to 430 °C. The error bars denote a standard deviation of ±8% in the measured *β*_*l*_ values and was calculated directly from the line profiles using Digital Micrograph. This plot is consistent with the thermomagnetic behaviour of the SQUID measurements from the entire thin film of sample 2 shown in Fig. 1b. In a separate experiment, we observed hysteresis in saturated induction measured from a DW during heating to, and cooling from, 300 °C, that was also in good agreement with Fig. 1b.

**Figure 9:**
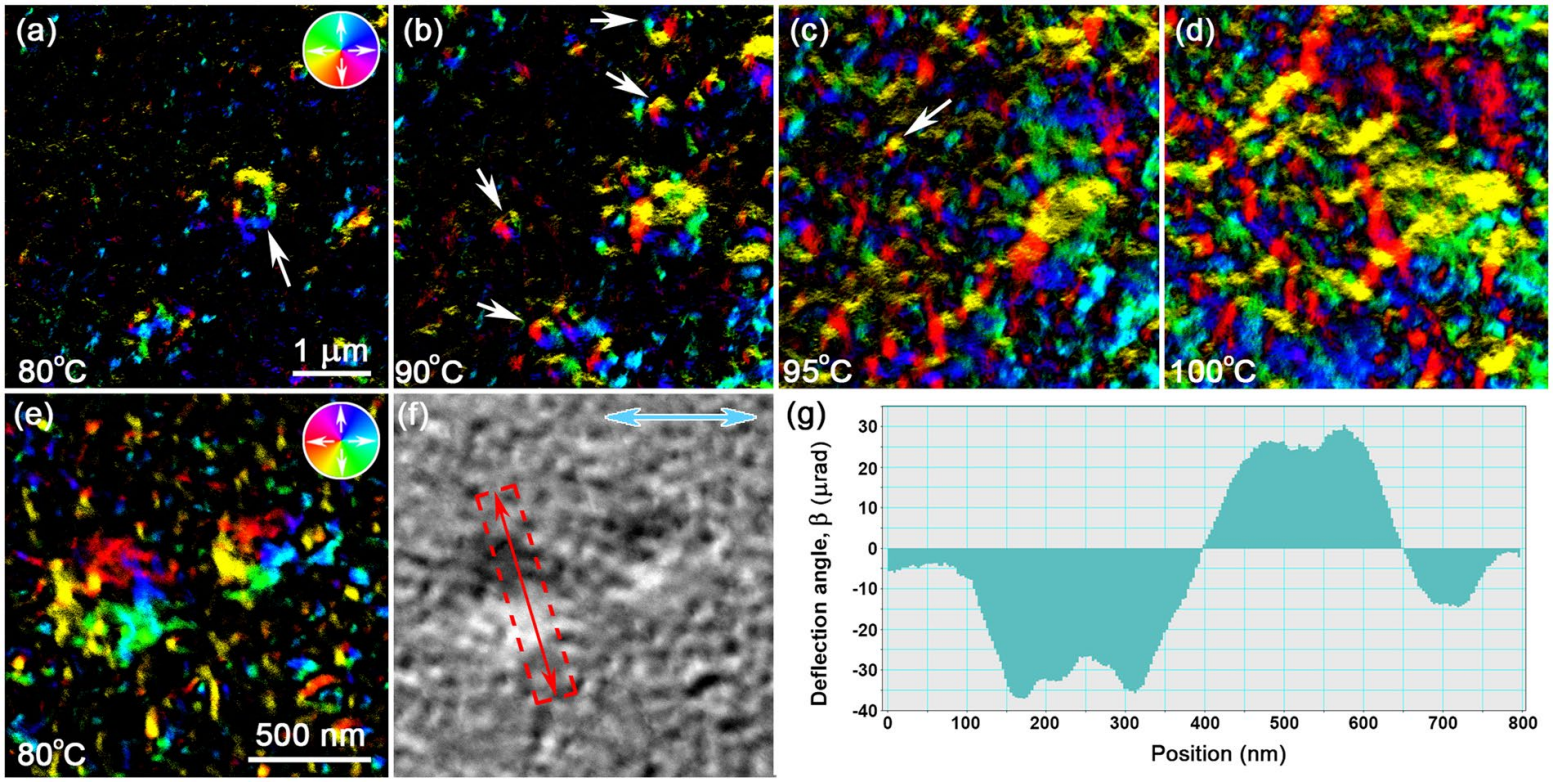
(**a**–**d**) DPC imaging of sample 3 during *in situ* heating, showing (**a**) presence of a magnetic domain structure at 80 °C (arrowed); (**b**) growth of magnetic domain in (**a**) and nucleation of vortex structures at 90 °C (arrowed); (**c**) nucleation of vortex structure (arrowed) and agglomeration into interacting magnetic strings at 95 °C; (**d**) formation of well-solved magnetic domains at 100 °C. (**e**) DPC image of small vortex structures at 80 °C and (**f**) an unsmoothed constituent component of magnetic induction, indicated by the double-headed blue arrows. (**g**) 40-line average profile taken across the red boxed region in (**f**).

Figure 9 presents the thermomagnetic behaviour of sample 3 as a function of temperature, providing information on the nucleation and growth dynamics of domains in the FeRh thin film as it undergoes its transition from AF to FM. Fast Fourier transform smoothing was performed to minimise the presence of high spatial frequency As-rich debris so that the DPC images are representative mainly of the magnetic induction in the FeRh thin film (described in more detail in the supplementary information (SI), Figs S1 and S2). The DPC image of Fig. 9a reveals the presence of a magnetic domain structure (arrowed) when heated to 80 °C, whilst most of the sample is considered to be in the AF state and are represented by the black regions, where no magnetic deflections are detected. The observed AF/FM boundary is similar to the edge of a FM nanostructure patterned in a non-magnetic film^20^ and hence the vortex state within the nanostructure is consistent with the reduction of magnetostatic energy at this boundary. This is also consistent with the circumstances that a nanoscale magnetic cylinder will form a vortex if it is thick enough and has a large enough diameter^30^. Micromagnetic modelling included in the SI (Fig. S3) further reinforces the energetics of the domain structure in this system of AF/FM coexistence at this early stage of the magnetic transition. As the FeRh thin film is heated to 90 °C (Fig. 9b), the magnetic domain highlighted in Fig. 9a is observed to grow and additional small vortex structures are seen to nucleate at scattered positions (arrowed). Heating to 95 °C (Fig. 9c) induces further nucleation of vortex structures (arrowed) and their agglomeration into an intricate system of interacting magnetic strings. The DPC image of Fig. 9d reveals the formation of well-resolved, uniformly-magnetised domains at 100 °C, which make up a complex magnetic network and is considered to be in a fully-FM state. The high magnification DPC image of Fig. 9e shows a localised view of the small vortex domain structures at 80 °C, surrounded by even smaller structures that are similar in size to the arsenic-rich surface debris. Figure 9f provides one constituent component of magnetic induction seen in Fig. 9e, indicated by the double-headed blue arrows, which allows for quantitative measurements. A 40-line average profile (boxed region, red) was taken across the vortex structure (Fig. 9f) and is displayed in Fig. 9g. The measured *β*_*l*_ is on a similar scale to those seen in Fig. 6g,h (~25 µrad) and equates to comparable value of *B*_s_ *t*. In a separate experiment involving cooling through the transition, we saw disintegration of the magnetic domains between 50 °C and 60 °C, which is consistent with hysteresis of 20 °C between heating and cooling shown in Fig. 1c.

## Discussion

This combined *in situ* TEM and DPC imaging study has provided a visual and quantitative investigation of the magnetostructural transition in planar FeRh thin films, prepared via FIB methods or HF-etching. Chemical analysis and HR imaging of cross-sections of samples 1 and 2 confirmed the epitaxial growth of single crystalline FeRh on the MgO substrate or NiAl buffer layer, respectively. In addition, SAED patterns from planar TEM lamellae of samples 1–3 further reinforced the evidence for the epitaxial growth of single crystalline FeRh, whilst their corresponding thickness profiles from EELS analysis revealed all samples were electron transparent. EDX chemical maps of sample 3 confirmed the HF-etching preparation produced a FeRh thin film and revealed small arsenic-rich debris dispersed across the sample, which were evident in the thickness profile of sample 3. Nevertheless, all three FeRh thin film samples were successfully prepared in planar view for TEM investigation of their magnetic behaviour using DPC imaging.

Initial DPC imaging of samples 1 and 2 (Fig. 5a,b) during *in situ* heating revealed complex systems of magnetic domains with varied directionality. Whilst the native magnetic state directly after preparation using FIB methods may represent a low energy configuration, it was difficult to isolate individual magnetic domains and perform quantitative measurements. Hence, it was necessary to uniformly magnetise the FeRh thin film whilst in the FM state; annihilating all the DWs by combining the use of an in-plane 50 mT magnetic field of the objective lens with *x*-tilting. Application of magnetic fields of increasing strength in the opposite direction then allowed for the nucleation and driving of DWs, providing a condition of parallel DWs considered favourable for quantitative measurements of *β*_*l*_. Direct measurements of *β*_*l*_ from the DWs as a function of temperature revealed contrasting thermomagnetic behaviour between sample 1 and 2. It is evident that sample 1 is strongly FM at 20 °C, with a calculated saturation induction of ~0.82 T, and its spontaneous magnetic induction steadily decreases with temperature as the FeRh film approached its *T*_*C*_, demagnetising at ~425 °C. In contrast, sample 2 is observed to be weakly FM at 20 °C, with a calculated saturation induction of ~0.21 T. A subsequent rapid increase in magnetic induction is seen from ~0.24 T at 60 °C to ~0.68 T at 100 °C, reaching a maximum of ~0.72 T at 120 °C and again steadily decreasing with increasing temperature, demagnetising at ~430 °C. The plot of saturation induction as a function of temperature in sample 2 is very similar to that of bulk measurements of equiatomic FeRh^3^, as demonstrated in Fig. 1b. Hence, it is considered the FIB sample preparation for TEM analysis has altered the FeRh of sample 1 to induce ferromagnetism, whereas the magnetostructural transition in sample 2 is somewhat preserved, with evidence of weak ferromagnetism at 20 °C.

Considering the main difference between the FeRh thin films in samples 1 and 2 are their respective substrate or buffer layer, it is necessary to consider the effect of the Ga^+^ ion beam during FIB preparation. The FeRh in sample 1 was grown epitaxially on MgO, a tough but brittle ceramic, and ion implantation has been shown to introduce defects in MgO in the form of high density microcracks^31^, resulting in a change in lattice parameter^32^. Further, strain in ultra-thin FeRh films (<10 nm) has been shown to alter the transition temperature^33^ and room temperature ferromagnetism at the interface between Rh-terminated surfaces of FeRh and MgO has been reported^34^. All these factors are considered to contribute to inducing ferromagnetism of the FeRh thin film observed in sample 1 through the FeRh-MgO interface. In contrast, the NiAl buffer layer is not grown epitaxially on the GaAs substrate, which is considered markedly soft relative to MgO, and hence Ga^+^ ion irradiation of the GaAs would have minimal effect on the interplanar spacing of the NiAl through the non-epitaxial interface, allowing the FeRh to mostly retain its magnetostructural transition. However, the residual weak FM moment at 20 °C can be attributed to clamping of the FeRh lattice at the interface with NiAl buffer layer^35^ (seen in Fig. 1b,c), as well as experiencing a degree of Ga^+^ irradiation, which has been shown to modify the magnetic properties of FeRh^36^, and would affect both samples 1 and 2.

The magnetostructural transition was most clear in sample 3, where fine details of the nucleation and evolution of magnetic domains with temperature were easily interpreted, due to the HF-etching of the substrate not noticeably affecting the integrity of the FeRh thin film. The DPC imaging suggests the transition from AF to FM regions occurs through nucleation of small vortex structures, which then grow through combining with newly nucleated vortex states via uniformly magnetised strings into larger complex magnetic domains. As temperature increases the magnetic network evolves into a system of generally larger uniformly-magnetised domains, where the FeRh is considered to be in a fully-FM state. Measurements of *β*_*l*_ from vortex structures in sample 3 were acquired at lower temperatures (80 °C), but it would be challenging to quantify the transition in a similar fashion as samples 1 and 2 due to the dynamic nature of the nucleation, growth and agglomeration of the domains and their DWs. For this reason, it could be said that the FIB damage that induced a small amount of FM in sample 2 facilitated a more accessible form of monitoring the magnetostructural transition from an individual DW, at temperatures considered below the transition seen in the bulk equivalent^3^. Nevertheless, the HF-etching and dynamic evolution of the magnetism in sample 3 is recognised as the most promising candidate for a more in-depth and localised examination of the true complexities of the magnetostructural transition in FeRh thin films. Further, due to the higher resolution of DPC imaging, this study has confirmed that an advanced new level of detail in understanding the magnetic transition in FeRh is achieved in comparison to other imaging techniques, such as XMCD-PEEM and SEMPA.

## Methods

### Electron microscopy

All the imaging, diffraction and spectroscopy described in this paper were carried out on a JEOL Atomic Resolution Microscope (JEM-ARM200F) TEM, operating at 200 kV^20^. This microscope is equipped with a cold field emission gun and a CEOS (Corrected Electron Optical Systems GmbH) probe corrector for STEM imaging. Conventional and HR STEM imaging were performed on cross-sectional and planar TEM samples of the FeRh films/substrates, whilst SAED acquired in TEM mode provided structural information. Both EDX and EELS provided chemical analysis of the samples. The sample thickness was determined by the spectrum imaging technique in STEM mode^37^, whereby low-loss EELS spectra acquired from each pixel were used to calculate values of t/λ. These calculations were performed using the Digital Micrograph^TM^ software package. The mean free path, λ, was determined from the density of equi-atomic FeRh and the TEM beam conditions, *i.e*., accelerating voltage, convergence and divergence angles, *etc*. and substituted into t/λ to calculate the relative thickness repetition, with a standard deviation of 6%. The magnetic structure of the FeRh films was visualised using DPC in Lorentz mode under low-magnetic field conditions or with an *in situ* applied field. DPC imaging was carried out with the HR objective lens pole piece switched off, with the samples positioned in the low-strength remanent field of the lens (~150 Oe out of plane). An 8-segment silicon photodiode array detector (supplied by DebenUK Ltd) was used for the DPC imaging. The signal from the detectors was converted and amplified using the “Superfast” amplifier (Andrew Armit Designs). The 8 detected signals were acquired, mixed and displayed via four Gatan DigiscanII units. In addition, combining DPC with *in situ* heating using the DENSsolution e-chips (up to 430 °C) or Gatan heating holder (up to 150 °C) allowed for direct access to the thermomagnetic behaviour of the DWs and magnetostructural transition within the FeRh films.

### Magnetic measurements

For comparison with bulk magnetic measurements from the entire thin films, thermomagnetic analysis of the FeRh samples was performed in a SQUID-VSM. These measurements were acquired by applying an in-plane saturating field and measuring the magnetic moment as a function of temperature.

### Data and materials availability

All data needed to evaluate the conclusions of this study can be found the at the following link 10.5525/gla.researchdata.558.

## Electronic supplementary material

Supplementary information
