# Supplementary material for: Quantitative TEM imaging of the magnetostructural and phase transitions in FeRh thin film systems
Source: Sci Rep. 2017 Dec 19;7:17835. doi: 10.1038/s41598-017-18194-0 (PMC5736605; doi:10.1038/s41598-017-18194-0)
Supplement: Supplementary file 1 — Supplementary information [file 41598_2017_18194_MOESM1_ESM.pdf]

Quantitative TEM imaging of the magnetostructural and phase transitions in FeRh thin film systems

Trevor P. Almeida[1]*, Rowan Temple[2], Jamie Massey[2], Kayla Fallon[2], Damien McGrouther[1], Thomas Moore[2], Christopher H. Marrows[2], Stephen McVitie[1]

[1] SUPA, School of Physics and Astronomy, University of Glasgow, Glasgow, G12 8QQ, UK.
[2]School of Physics and Astronomy, University of Leeds, LS2 9JT, UK.

Supplementary information

*Corresponding author:
Tel: +44 (0) 141 330 2879
Email: trevor.almeida@glasgow.ac.uk

This PDF file includes:
Fast Fourier Transform smoothing of DPC images
Micromagnetic Modelling

Fast Fourier Transform smoothing of DPC images

The DPC images of the HF-etched FeRh sample underwent a series of smoothing steps to isolate the magnetic induction from the high spatial frequency artefacts and present a state of AF / FM co-existence. Figures S1 summarises the initial processing steps with the description on the left-hand side.

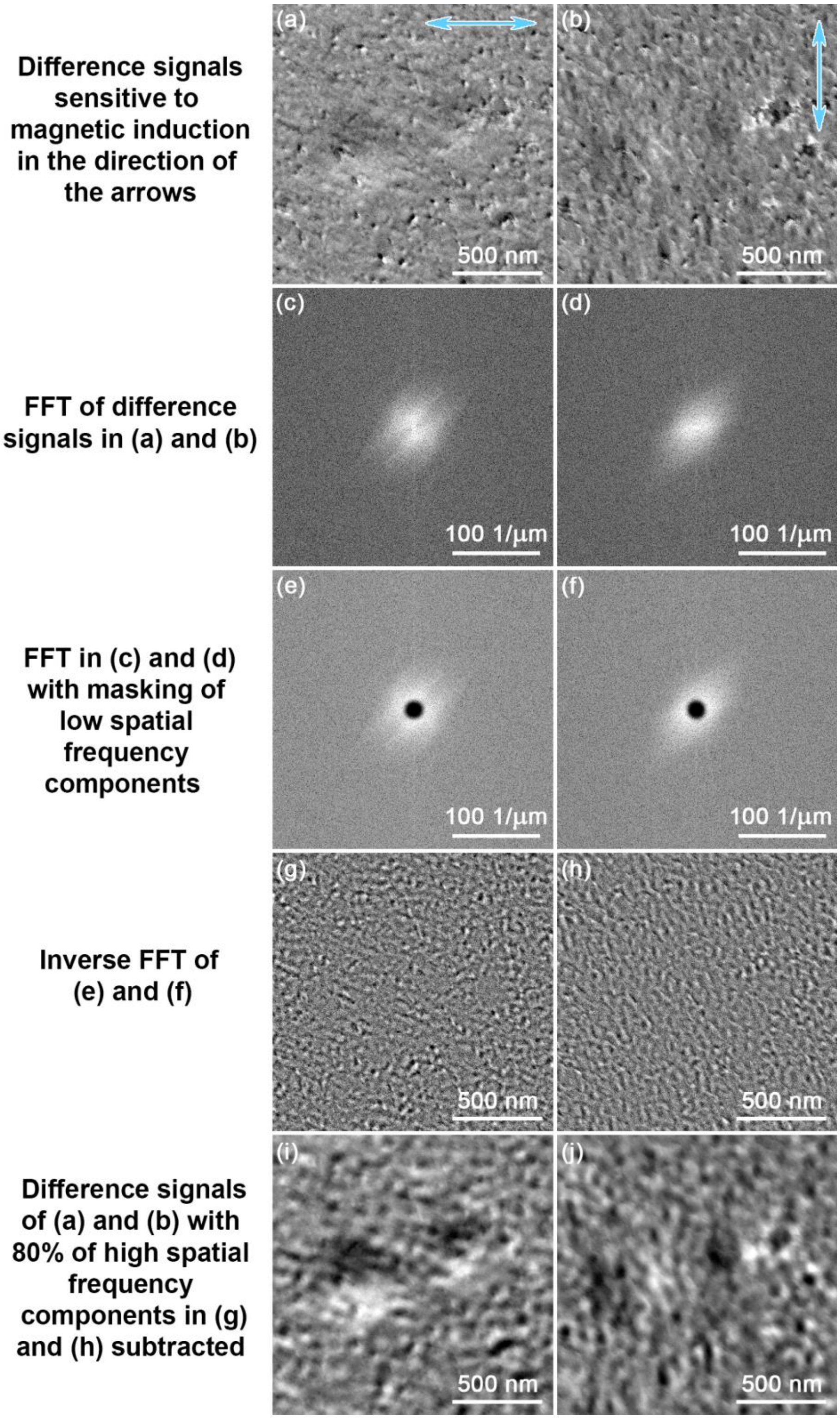

Figure S1. Processing steps of (a,b) difference signals of DPC image. (c,d) FFT of difference signals in (a,b); and (e,f) masking (~ 30 1/μm in diameter) of low spatial frequency components. (g,h) Inverse FFT of (e,f) showing the high spatial frequency components. (i,j) Smoothed difference signals with subtraction of 80% high spatial frequency components.

Once smoothed, the difference signals are combined to illustrate the direction of magnetic induction using colour (Fig. S2a). The colour image is then combined with the normalised magnitude image of the difference signals (Fig. S2b) to create a magnetic induction map of FeRh in a state of AF / FM coexistence (Fig. S2c).

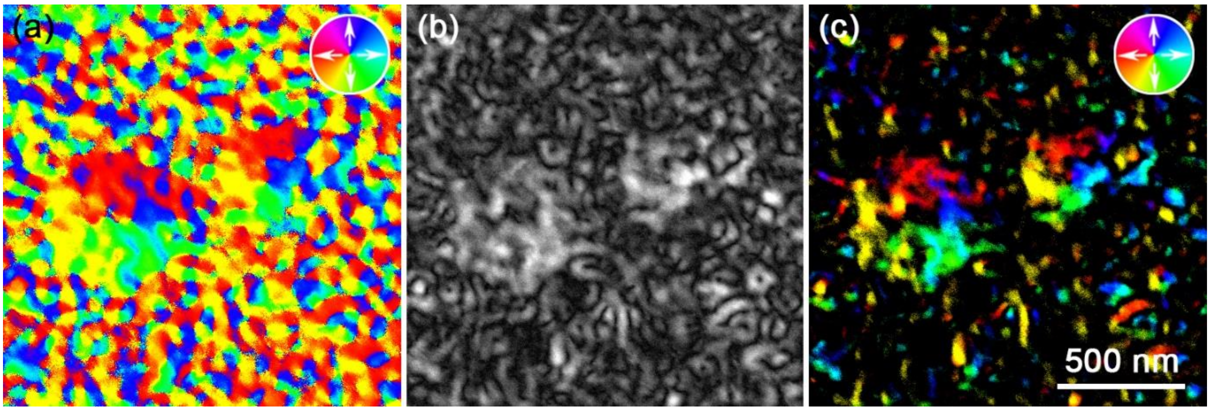

Figure S2. (a) Colour image of the difference signals showing the direction of magnetic induction. (b) Normalised magnitude image of the difference signals. (c) Magnetic induction map showing vortex structures within the FeRh in a state of AF / FM coexistence.

Micromagnetic modelling

A micromagnetic simulation was performed using the GPU-accelerated mumax^3 software[1]. The simulation environment has dimensions of 6 µm by 6 µm in-plane with a thickness of 50 nm and a cell size of 5 nm×5 nm×50 nm. The micromagnetic parameters used were $A_{ex} = 13\times10^{-12}$ Jm^{-1}, $M_s = 860\times10^3$ Am^{-1} and no magnetocrystalline anisotropy. The simulation was constructed with four distinct cylinders with diameters ranging from 200 nm to 400 nm and with saturation magnetisation M_s. The cylinders were surrounded by a continuous background region with saturation magnetisation 0.2 M_s (Fig S3d). All other micromagnetic parameters were held constant between the regions. The simulation was allowed to relax from uniformly magnetisation in the +*x* direction. The magnetisation components resulting from this simulation are shown in figures S3a-c. As can be seen in figures S3a-c the system relaxed into a ground state with vortex states in each of the cylinders, due to the significant magnetostatic energy generated at the boundary between the full M_s and partial M_s regions. For comparison to the experimental images, a simulated DPC image (Fig S3e) was calculated from the magnetisation components. This was achieved by calculating the *x* and *y* components of the integrated magnetic induction (the electrons were assumed to be travelling in a trajectory normal to the sample surface) and combining these into a colour image following the same image processing steps outlined above. Figure S3e shows magnetic contrast comparable to the domain structure seen in figure 8b of the main text.

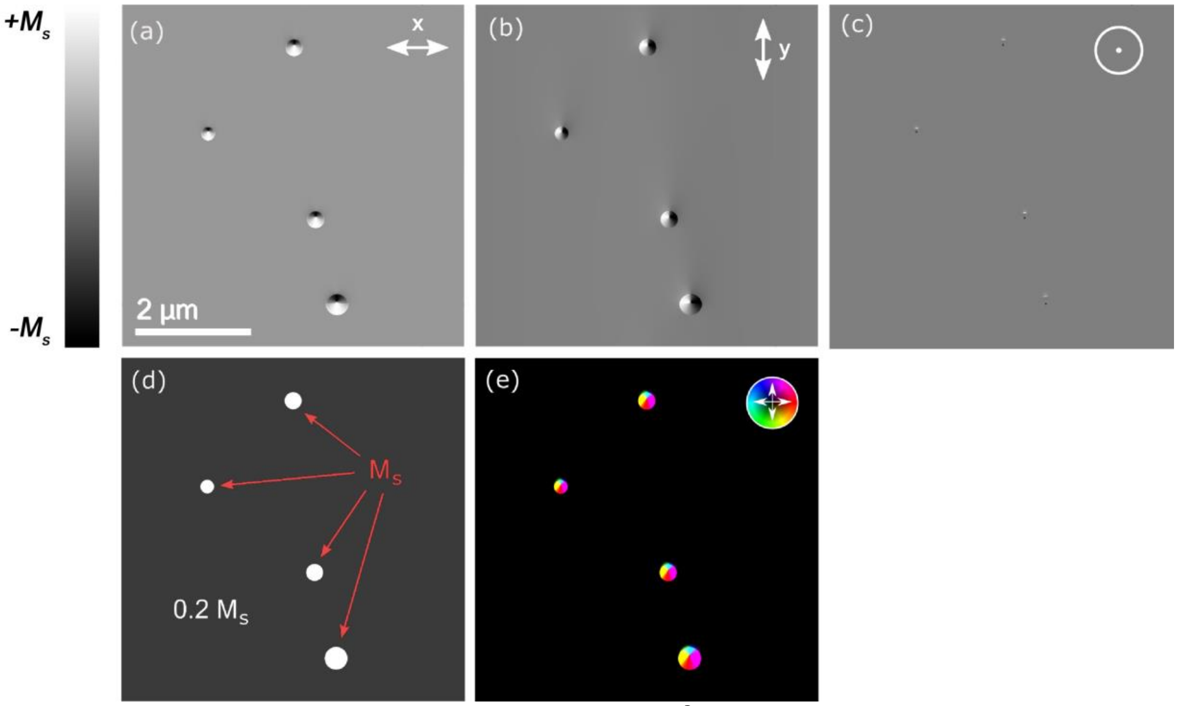

Figure S3. (a-c) M_x, M_y and M_z, respectively, as output by mumax^3. (d) The initial state of the simulation, where white indicates areas with the full saturation magnetisation, M_s, and dark grey indicates areas with partial saturation magnetisation, 0.2M_s. (e) DPC image calculated from the magnetisation components in (a)-(c). The direction of the magnetic induction is given by the colour wheel.

References

1. A. Vansteenkiste, J. Leliaert, M. Dvornik, M. Helsen, F. Garcia-Sanchez, B. Van Waeyenberge, "The design and verification of mumax3", AIP Advances 4 (2014) 107133.
